# Lateralization of cerebral blood flow in the auditory cortex of patients with idiopathic tinnitus and healthy controls: An arterial spin labeling study

**DOI:** 10.3389/fnins.2022.992758

**Published:** 2022-12-07

**Authors:** Xiaoshuai Li, Yansheng Zhao, Ying Hui, Yuntao Wu, Qian Chen, Huijing Shi, Han Lv, Mengning Li, Pengfei Zhao, Wenfei Zhang, Xinyu Zhao, Jing Li, Liufu Cui, Zhenchang Wang

**Affiliations:** ^1^Department of Radiology, Beijing Friendship Hospital, Capital Medical University, Beijing, China; ^2^Department of MRI Room, Kailuan General Hospital, Tangshan, Hebei, China; ^3^Department of Cardiology, Kailuan General Hospital, Tangshan, Hebei, China; ^4^Department of Rheumatology and Immunology, Kailuan General Hospital, Tangshan, Hebei, China; ^5^Clinical Epidemiology and Evidence-Based Medicine Unit, Beijing Friendship Hospital, Capital Medical University, Beijing, China

**Keywords:** tinnitus, auditory cortex, arterial spin labeling, cerebral blood flow, lateralization

## Abstract

**Objectives:**

To assess the lateralization of cerebral blood flow (CBF) in the auditory cortex of idiopathic tinnitus patients and healthy controls (HCs) using 3D pseudocontinuous arterial spin labeling (pcASL).

**Methods:**

Thirty-six patients with idiopathic tinnitus and 43 sex- and age-matched HCs underwent 3D-pcASL scanning using a 3.0 T MRI system. For both groups, region of interest analysis was performed on the primary auditory cortex (PAC), auditory associative cortex (AAC), and secondary auditory cortex (SAC). The clinical data of all subjects were analyzed.

**Results:**

In both tinnitus patients and HCs, CBF of the left PAC was significantly higher than that of the right (HCs: *P* = 0.02; patients: *P* = 0.043), but CBF of the right AAC and SAC was significantly higher than that of the left (AAC: HCs, *P* < 0.001; patients: *P* < 0.001. SAC: HCs, *P* < 0.001; patients: *P* = 0.001). Compared with HCs, tinnitus patients exhibited significantly higher CBF in the bilateral PAC (right: *P* = 0.008; left: *P* = 0.022). CBF in the left PAC was positively correlated with tinnitus severity (*r* = 0.399, *P* = 0.016).

**Conclusion:**

This study confirms the asymmetry of the auditory cortex and investigates the underlying neuropathology of idiopathic tinnitus in terms of CBF.

## Introduction

Idiopathic tinnitus is a subjective auditory perception that occurs in the absence of external sound stimuli. In adults, the incidence of idiopathic tinnitus is as high as 15% and increases with age; consequently, idiopathic tinnitus has become a major health issue worldwide (Conlon et al., 2020; Henton and Tzounopoulos, 2021). This condition seriously affects quality of life and can lead to depression, sleep disturbance, anxiety and even suicide.

The etiology of idiopathic tinnitus remains unclear. It is considered to originate from abnormal neuronal activity in the auditory cortex (AC) (Hayes et al., 2021). In animal models of tinnitus, auditory cortical neurons exhibit increased spontaneous firing rates (Basura et al., 2015; Takacs et al., 2017), and functional magnetic resonance imaging (fMRI) has revealed similar findings in the human AC (Chen et al., 2017; Han et al., 2018). The AC is one of the most plastic cerebral cortexes and is involved in the processing of sound information. Long-term abnormal neural activity can cause structural and functional plasticity of the AC, resulting in persistent tinnitus (Adjamian et al., 2014). The inhibitory gating mechanism, a system that prevents tinnitus signals from reaching the AC, is also thought to be related to the development of tinnitus (Rauschecker et al., 2010). Under normal conditions, abnormal neural activity associated with tinnitus is counteracted at the thalamic level by inhibitory feedback loops from the limbic system. However, compromise of the inhibitory gating mechanism results in transmission of abnormal activity to the AC and its perception as tinnitus (Adjamian et al., 2014). Therefore, the AC plays a key role in the mechanism of tinnitus.

Currently, the lateralization of the AC in idiopathic tinnitus patients remains unclear. Geven et al. (2014) used positron emission tomography (PET) to describe the lateralized activation of the AC in tinnitus patients; the primary AC (PAC) undergoes leftward lateralization, while the auditory associative cortex (AAC) and secondary AC (SAC) undergo rightward lateralization. However, the sample size of the study by Geven et al. was small, and no statistical analysis of activation of the bilateral AC was performed to provide additional evidence. Several fMRI studies of sound-evoked neuronal activity in tinnitus patients have demonstrated lateralized activation of the AC (Smits et al., 2007; Lanting et al., 2014). But, experimental auditory stimuli may interfere with resting-state activity of the AC. Thus, quantitative assessment of lateralization in the AC in tinnitus at rest is an important topic.

Neuronal activity is closely coupled to cerebral blood flow (CBF), and increased neuronal activity leads to increased CBF (Raichle and Mintun, 2006; Lanting et al., 2009). Arterial spin labeling (ASL) can non-invasively measure CBF by using endogenous arterial blood protons as contrast agents. Compared with PET, computed tomography perfusion, and dynamic contrast-enhanced MRI, ASL is non-invasive, simple, and has high repeatability. Moreover, the accuracy of this technique has been verified (Dolui et al., 2020). Due to its increased signal-to-noise ratio, 3D pseudocontinuous ASL (pcASL) is widely used to identify CBF changes under different pathological conditions, including tinnitus (Li et al., 2020, 2021; Xia et al., 2020).

Thus, the aim of this study was to assess the lateralization of CBF in the AC in idiopathic tinnitus patients and healthy controls (HCs) using 3D-pcASL. In addition, we explored the differences in CBF in the AC between patients with tinnitus and HCs, and the correlation of altered CBF with clinical data.

## Materials and methods

### Subjects

This research was approved by the Medical Research Ethics Committees. Written informed consent was acquired from each participant in accordance with the Declaration of Helsinki.

In this study, 36 bilateral tinnitus patients and 43 sex- and age-matched HCs were enrolled from Kailuan community. Patients were included if the following criteria were met: persistent idiopathic tinnitus, self-reported normal audiology, no prior history of central nervous system disorders or head trauma, and no MRI contraindications. Subjects were excluded if they had pulsatile tinnitus, hyperacusis, prior stroke, systemic diseases, or a history of drug or alcohol abuse in the previous 3 months. Tinnitus is divided into 6 grades according to its severity: grade 1, in which tinnitus is seemingly non-existent; grade 2, in which tinnitus is only experienced in quiet environments; grade 3, in which tinnitus is experienced in daily environments and does not affect quality of life; grade 4, in which tinnitus is experienced in noisy environments and affects sleep; grade 5, in which tinnitus seriously affects sleep and work; and grade 6, in which tinnitus leads to anxiety and depression.

### Magnetic resonance imaging scanning

All subjects were scanned on a GE 750W 3.0 T MR scanner (General Electric, Milwaukee, WI, USA). We used earplugs and foam padding to reduce scanner noise and head motion, respectively. All participants were instructed to close their eyes and avoid thinking of anything. Structural images were obtained using a brain volume sequence (echo time (TE) = 2.8 ms; inversion time (TI) = 450 ms; repetition time (TR) = 7.5 ms; flip angle (FA) = 15°; slice thickness = 1 mm; matrix = 256 × 256; and field of view (FOV) = 256 mm × 256 mm). Perfusion data were acquired by a 3D-pcASL sequence (TE = 10.7 ms; TR = 5313 ms; slice thickness = 4 mm; post-label delay (PLD), 2525 ms; and FA = 111°; FOV, 240 mm × 240 mm; and in-plane resolution, 3.37 × 3.37 mm). The acquisition time of ASL was 4 min and 45 s.

### Data preprocessing

In the AW4.6 workstation, the ASL difference images were derived by pairwise subtraction of the control and label images; subsequently, the CBF map was obtained from the ASL difference images. Details of the processing procedures were described in a previous study (Li et al., 2022). The CBF maps of all patients and HCs were coregistered to MNI space using SPM8 software. Subsequently, we standardized the CBF of each voxel by dividing it by the mean CBF of the whole brain (Aslan and Lu, 2010). Finally, the 8-mm FWHM Gaussian kernel was used to smooth the standardized CBF maps.

**FIGURE 1:**
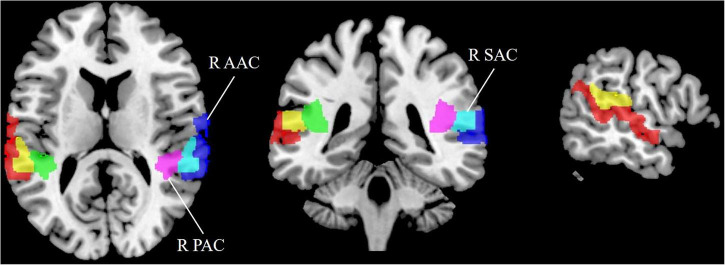
Region of interest (ROI) analysis for quantitative evaluation of CBF in the auditory cortex. SAC, secondary auditory cortex; AAC, auditory associative cortex; PAC, primary auditory cortex; CBF, cerebral blood flow; R, right; L, left.

Tinnitus has been confirmed to originate from abnormal neuronal activity in the AC (Hayes et al., 2021). Thus, we performed ROI analysis of the AC in tinnitus patients, including the AAC, PAC, and SAC (Figure 1). Brodmann’s area (BA) template was used to extract the bilateral AAC (BA22), PAC (BA41), and SAC (BA42). The CBF in each ROI in tinnitus patients and HCs was extracted based on the above templates.

The lateralization index (LI), which was calculated as: (left–right)/(left+right) (Seghier, 2008), was used to assess the symmetry of CBF in the bilateral AC. The LI ranged between −1 and 1. A negative LI represented higher CBF in the right AC, and a positive LI indicated higher CBF in the left AC.

### Statistical analysis

The chi-square test and two-sample *t*-test were performed to identify group differences in demographic data. CBF differences in the bilateral AC of each group were explored using the paired samples *t*-test. We used one-sample *t*-test as the comparison point to investigate the LI difference in the AC of each group. The analysis of covariance was used to compare the difference in CBF and LI in each AC between patients and HCs, with age and sex as covariates. In addition, we analyzed the correlations of altered CBF with the duration and severity of tinnitus. All results were presented without correction for multiple comparisons.

## Results

### Sample characteristics

The baseline information for all subjects is shown in Table 1. There were no significant differences in age, sex, and handedness between the two groups. The duration of tinnitus was 41.3 ± 24.6 months. There were 6 patients with grade 1, 10 patients with grade 2, 9 patients with grade 3, 5 patients with grade 4, and 6 patients with grade 5.

**TABLE 1:** Sample characteristics of patients with tinnitus and HCs.

	Tinnitus (*n* = 36)	HC (*n* = 43)	*P*
Age (years)	54.1 ± 10.2	51.3 ± 8.3	0.181^a^
sex (male/female)	12/24	13/30	0.768^b^
Tinnitus side	Bilateral	–	–
Handedness	36 right-handed	43 right-handed	1.000^a^
Tinnitus duration (months)	41.3 ± 24.6	–	–

^a^Two-sample *t*-test.

^b^chi-square test; HC, healthy control.

### Cerebral blood flow analysis

**FIGURE 2:**
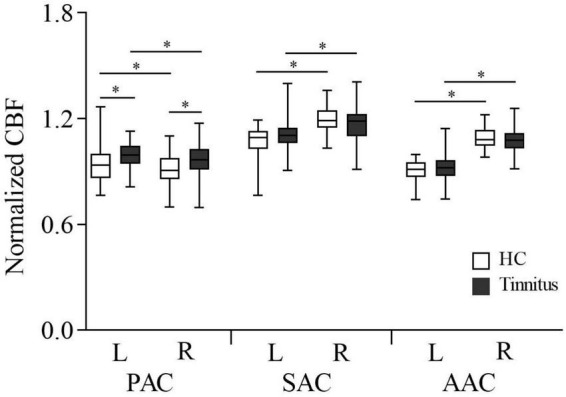
Normalized CBF according to ROI analysis of the auditory cortex in tinnitus patients and HCs. AAC, auditory associative cortex; SAC, secondary auditory cortex; PAC, primary auditory cortex; CBF, cerebral blood flow; HC, healthy control; ROI, region of interest; L, left; R, right. *Statistical significance.

The CBF in the AC in each group of subjects is shown in Figure 2. In both HCs and tinnitus patients, CBF of the left PAC was significantly higher than that of the right (HCs: 0.942 ± 0.102 vs. 0.911 ± 0.089, *P* = 0.02; patients: 0.996 ± 0.077 vs. 0.969 ± 0.089, *P* = 0.043), but CBF of the right AAC and SAC was significantly higher than that of the left (AAC: HCs, 0.908 ± 0.057 vs. 1.093 ± 0.066, *P* < 0.001; patients: 0.919 ± 0.079 vs. 1.073 ± 0.071, *P* < 0.001. SAC: HCs, 1.075 ± 0.084 vs. 1.193 ± 0.074, *P* < 0.001; patients: 1.109 ± 0.101 vs. 1.163 ± 0.096, *P* = 0.001). In addition, compared with HCs, patients with tinnitus exhibited significantly higher CBF in the bilateral PAC (right: 0.969 ± 0.089 vs. 0.911 ± 0.089, *P* = 0.008; left: 0.996 ± 0.077 vs. 0.942 ± 0.102, *P* = 0.022).

**FIGURE 3:**
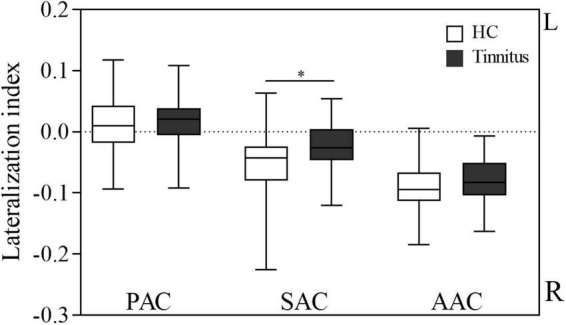
Lateralization index of cerebral blood flow in the auditory cortex in the two groups. AAC, auditory associative cortex; SAC, secondary auditory cortex; PAC, primary auditory cortex; HC, healthy control; R, right; L, left. *Statistical significance.

Figure 3 shows the LI of the AC in the two groups. CBF was lateralized to the left in the PAC and lateralized to the right in the SAC and AAC in both tinnitus patients and HCs. One-sample *t*-test showed that in each group, the LI of the PAC was significantly higher than 0 (HCs: *P* = 0.023; patients: *P* = 0.037), while the LI of the SAC and AAC were significantly lower than 0 (SAC: HCs, *P* < 0.001; patients, *P* = 0.001; AAC: HCs, *P* < 0.001; patients, *P* < 0.001). In addition, tinnitus patients had significantly lower LI in SAC than HCs (*P* = 0.008), and no significant LI difference was found in PAC and AAC between the two groups (*P* > 0.05).

### Correlation analysis

**FIGURE 4:**
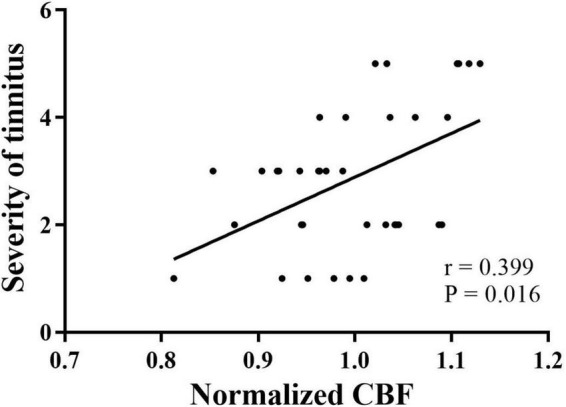
Correlation between the normalized CBF of the left PAC and severity of tinnitus in patients with tinnitus. PAC, primary auditory cortex; CBF, cerebral blood flow. ^•^Grades of tinnitus severity.

Spearman correlation analysis showed that in tinnitus patients, CBF in the left PAC was positively correlated with the severity of tinnitus (*r* = 0.399, *P* = 0.016) (Figure 4). No significant correlations were found between altered CBF in the other AC and the clinical data.

## Discussion

This study investigated the lateralization of CBF in the AC of patients with idiopathic tinnitus using the 3D-pcASL technique. CBF was asymmetric in the AC in both tinnitus patients and HCs. Moreover, CBF in the bilateral PAC was significantly higher in tinnitus patients than in HCs. The higher CBF in the left PAC was positively correlated with tinnitus severity. These findings contribute toward an understanding of the neuropathological mechanisms of tinnitus in terms of CBF.

The AC is responsible for processing auditory information. A previous study demonstrated that the columnar and connective structures of the AC in the left cerebral hemisphere differ anatomically from those in the right hemisphere (Hutsler and Galuske, 2003). Lanting et al. (2014) used fMRI and found that compared with HCs, tinnitus patients showed smaller lateralization for sound stimuli, reaching significance in the right PAC. However, auditory stimuli may interfere with resting-state neural activity in the AC. Geven et al. described the asymmetry of metabolic activity in the AC of tinnitus patients using the PET technique. They found that in both tinnitus patients and HCs, the activity in the right AAC and SAC was higher than that in the left, while the activity in the left PAC was higher than that in the right (Geven et al., 2014). However, because Geven et al. did not investigate significant differences in metabolic activity in the bilateral AC, there is no strong evidence that AC activity is asymmetric.

In the present study, we compared CBF differences in bilateral AC using the 3D-pcASL technique and found that the CBF in the right AAC and SAC was significantly higher than that in the left, while the CBF in the left PAC was significantly higher than that in the right. We also used the LI to confirm the asymmetry of CBF in the AC. These findings suggest that asymmetric CBF in the bilateral AC is a physiological feature of both tinnitus patients and HCs, regardless of the presence or absence of tinnitus.

The CBF in the bilateral PAC was significantly higher in tinnitus patients than in HCs in this study. Auditory stimuli can increase neural activity in the bilateral AC. De Ridder et al. (2007) found a relationship between the degree of PAC reorganization and tinnitus intensity. Previous studies have suggested that tinnitus originates from abnormal activity in the PAC due to hearing loss (Roberts et al., 2013; Roberts, 2018). Since neuronal activity is closely coupled to CBF, it is reasonable that patients with tinnitus have significantly higher CBF in the bilateral PACs. In addition, CBF in the left PAC was positively correlated with the severity of tinnitus. We speculate that tinnitus patients with higher CBF in the left PAC may have greater difficulty adapting to tinnitus and therefore have higher grade. The correlation between the change in CBF in the left PAC and tinnitus severity needs to be studied further to verify this hypothesis.

Several limitations of this study should be noted. The sample size of this study was limited, and more patients need to be enrolled in the future to further verify these results. Second, all patients in this study had bilateral tinnitus, which is the most common type of tinnitus observed in clinical practice. In future work, we will include patients with unilateral tinnitus and evaluate lateralization in the AC. Third, MRI noise may have a potential effect on the results. In this study, we used earplugs and foam pads to reduce scanner noise, thereby minimizing the impact of noise on the results. In the future, we will further explore the effect of noise on CBF in AC. In addition, we will recruit left-handed subjects to eliminate the possible effect of handedness on the results.

## Conclusion

In this study, the asymmetry of the AC in tinnitus patients and HCs was identified using the 3D-pcASL technique. CBF in the PAC was left-lateralized in all subjects, and CBF in the SAC and AAC was right-lateralized. Moreover, CBF in the bilateral PAC was higher in tinnitus patients than in HCs. In this study, we not only investigated the underlying neuropathology of tinnitus in terms of CBF but also may have implications for the rationale of tinnitus treatment protocols based on lateralized hyperactivity.

## Data availability statement

The raw data supporting the conclusions of this article will be made available by the authors, without undue reservation.

## Ethics statement

The studies involving human participants were reviewed and approved by the Ethics Committee of Beijing Friendship Hospital. The patients/participants provided their written informed consent to participate in this study.

## Author contributions

XL, YZ, YH, QC, HS, ML, and WZ performed the experiment and collected, analyzed, or interpreted the data involved in the study. XL preprocessed image data, performed the statistical results, and drafted the manuscript. PZ, HL, XZ, and YW designed the study and ensured the questions related to all aspects of the work. JL, LC, and ZW gave critical comments on the manuscript. All authors contributed to the article and approved the submitted version.
